# Role of Phosphorylation in the Modulation of the Glucocorticoid Receptor’s Intrinsically Disordered Domain

**DOI:** 10.3390/biom9030095

**Published:** 2019-03-11

**Authors:** Raj Kumar, E. Brad Thompson

**Affiliations:** 1 Department of Medical Education, Geisinger Commonwealth School of Medicine, Scranton, PA 18509, USA; 2 Department of Biochemistry and Molecular Biology, University of Texas Medical Branch, Galveston, TX 77555, USA

**Keywords:** glucocorticoid receptor, phosphorylation, intrinsically disordered, transactivation activity, gene regulation, coactivators

## Abstract

Protein phosphorylation often switches cellular activity from one state to another, and this post-translational modification plays an important role in gene regulation by the nuclear hormone receptor superfamily, including the glucocorticoid receptor (GR). Cell signaling pathways that regulate phosphorylation of the GR are important determinants of GR actions, including lymphoid cell apoptosis, DNA binding, and interaction with coregulatory proteins. All major functionally important phosphorylation sites in the human GR are located in its N-terminal domain (NTD), which possesses a powerful transactivation domain, AF1. The GR NTD exists as an intrinsically disordered protein (IDP) and undergoes disorder-order transition for AF1’s efficient interaction with several coregulatory proteins and subsequent AF1-mediated GR activity. It has been reported that GR’s NTD/AF1 undergoes such disorder-order transition following site-specific phosphorylation. This review provides currently available information regarding the role of GR phosphorylation in its action and highlights the possible underlying mechanisms of action.

## 1. Introduction

**Figure 1:**
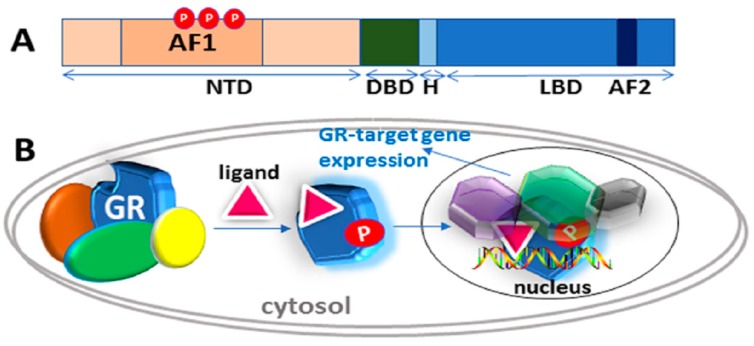
Classical action of the glucocorticoid signaling mediated by the glucocorticoid receptor (GR). (**A**) A topological diagram of human GR protein showing major functional domains and major known AF1 phosphorylation (P) sites (other GR sites not shown) [6]. NTD, N-terminal domain; DBD, DNA binding domain; H, Hinge region, LBD, Ligand binding domain. (**B**) Unliganded receptor is located in the cytosol associated with several heat shock and other chaperone proteins including HSP90, HSP70, CyP-40, P23, and FKBPs (shown by different colors around GR). Ligand binding leads to conformational alterations in the GR, and by doing so GR dissociates from these associated proteins, and ligand bound GR is free to translocate to the nucleus. This process appears to be phosphorylation (P) dependent. Once in the nucleus, GR binds to site-specific DNA binding sequences and interacts with several other coregulatory proteins (shown by different colors and shapes around GR), and subsequently leads to transcriptional regulation. Based on reference [10].

The glucocorticoid receptor (GR) is a well-known, ligand-driven transcription factor, essential for many of the functions-physiologic, pathological, and therapeutic of hormonal and synthetic glucocorticoids [1,2,3,4,5,6,7,8]. The GR belongs to the superfamily of the steroid and thyroid hormone-activated intracellular transcription factors, and the larger family of nuclear hormone receptors (NHRs) [9,10,11,12,13]. The GR was the first member of this superfamily to be cloned and characterized [14]. It is a ubiquitously expressed intracellular protein that regulates the expression of glucocorticoid-responsive genes in a cell/tissue- and promoter-specific manner [9,10]. The broad overview of glucocorticoid action (Figure 1) states that the cytosolic GR is part of a large heteromeric complex consisting of several chaperone proteins including HSP90, HSP70, p23, immunophilins of the FK506-binding protein family (FKBP51 and FKBP52) and possibly several others [15,16,17,18]. These proteins maintain the receptor in a transcriptionally inactive conformation that favors high affinity ligand binding [15,16,17,18].

Glucocorticoid binding to GR’s C-terminal ligand binding pocket leads to structural rearrangements, causing the receptor to be released from the complex. At some point, the GR becomes hyper-phosphorylated and active [15,16,17,18,19], enters the nucleus and interacts with site-specific DNA sequences, termed “glucocorticoid response elements” (GREs), and several additional coregulatory proteins. (Figure 1). The GR can also bind at heterodox regulatory elements by “piggybacking” on other transcription factors [10,11,13]. The DNA and protein interactions are highly dynamic in the genomic context, as the receptor rapidly moves from one site to another and interacts with various proteins [9]. One important implication of this model is that the surfaces of the GR must be employed in various ways in order to allow temporary interactions with a variety of other macromolecules, and thus change transcription [9]. In this review, we discuss the structure and functions of the GR, specifically the role of site-specific phosphorylation in the regulation of its intrinsically disordered (ID) NTD.

## 2. The Structure of the Glucocorticoid Receptor and its Gene

The human GR gene consists of 9 exons located on chromosome 5 [20,21]. Like other steroid hormone receptors (SHRs), the GR consists of three well-known major functional domains: N-terminal (NTD), DNA binding (DBD), and ligand-binding (LBD) (Figure 1A). DBD and LBD are separated by a short intrinsically disordered (ID) amino acid sequence known as the “hinge” region [13]. Within the NTD and LBD are two transcription activation function regions, AF1 and AF2, respectively [13]. AF2 is strictly ligand-dependent whereas AF1 is ligand-dependent in the context of the holo-GR but is constitutively active and can regulate GR-target genes in a ligand-independent manner when the LBD is removed [9,13]. In other words, the AF1 can act constitutively in the absence of the LBD and is quite active in stimulating transcription from simple promoters containing cognate GR binding sites [13]. With the discovery of a large cohort of GR forms with unique expression, gene-regulatory, and functional profiles [6], the traditional view that a single GR protein regulates the effects of glucocorticoids has changed in recent years. Alternative exon splicing and translation initiation sites in the human GR mRNA result in a number of receptor sub-types [6].

When interacting with chromosomal DNA, both AF domains of the GR mediate transcriptional activation by recruiting coregulatory multi-subunit complexes that remodel chromatin, target initiation sites, and stabilize the RNA polymerase II machinery for repeated rounds of transcription of target genes [9]. In the conceptual model of receptor:coactivator complexes, the ligand-bound GR recruits one or more cofactors, which subsequently results in the recruitment of additional known cofactors to the assembly of the complex [9,13]. Depending on the GR ligand, these cofactors may lead to increased or reduced transcription of regulated genes. It is likely that additional as yet unknown cofactors are involved, and that different GRs may recruit different components to the complex, thus achieving a level of specificity among GRs and coactivators or corepressors [9].

Though the structures of independently expressed, more stably structured LBD and DBD were solved long ago [22,23], no 3D structure of full-length GR is currently known. From the LBD structural and mutation data, it is clear that ligand binding results in conformational rearrangement of AF2 sub-domain (usually helix 12) such that its surfaces are available for interactions with specific coregulatory proteins through LXXLL motifs [23]. Bound to an agonist ligand, AF2 adopts a conformation that suits for interaction with coactivators, whereas an antagonist binding blocks such interactions and rather opens surfaces for corepressor interactions [23]. However, many ligands originally labeled “antagonists” are actually weak partial agonists that compete for the LBD site. Presumably, when bound with these, the LBD-ligand-coactivator interactions are weaker, so that gene induction is reduced. Furthermore, the GR LBD crystal structure revealed a second charge clamp, which may determine the binding selectivity of a coactivator [23]. The structure of the DBD and how it fits into its GRE has been known for some time [22], though this binding process may be more dynamic than once envisaged [9].

Due to its disordered nature, our understanding about the structure and functions of AF1 has languished until recently. The GR AF1 supplies most of the transcription-controlling power of the GR; and this lack of information about how AF1 interacts with various coregulatory proteins, and the consequences for transcriptional regulation, has hampered understanding of the full spectrum of GR action. The AF1 activation domain was discovered well before AF2 and was initially thought to be the only GR transactivation function. The major obstacle in solving full-length GR structure or that of AF1 alone is the fact that large portion of the GR NTD, including AF1, is IDP [24,25,26,27,28,29,30]. A new, quantitative thermodynamic model for allosteric interdomain coupling has been proposed that explains the role of the IDP NTD of the GR in the receptor’s function [31]. This model would be applicable to other SHRs and transcription factors generally.

## 3. The IDP Nature of the GR NTD/AF1 Means that it Can be Thought of as a Large Ensemble of Rapidly Interchanging Conformations

Compared to the LBD and DBD, the GR NTD is most variable in terms of sequence homology and size among various mammals [10]. The AF1 plays an important role in the interaction of the receptor with molecules necessary for the initiation of transcription, such as chromatin modulators and protein from basal transcription factors, including RNA polymerase II, TATA-binding protein (TBP) and a host of TBP-associated proteins [9,28]. The AF1 is also known to interact with many other coregulatory proteins including coactivators and corepressors, which are essential for optimal GR activity in a cell/tissue-specific manner [9,10,12,28]. Several of these coregulatory proteins are also known to interact with the AF2 region [9,10,12,28]. However, unlike the LXXLL binding motif for AF2 interactions, no such motif is known for the AF1 [23], and in fact, IDP regions usually lack a defined interaction motif as in many transcription factors [32,33,34].

IDPs are subject to combinatorial alternative splicing and post-translational modifications, adding complexity to regulatory networks and providing a mechanism for cell/tissue-specific signaling [35,36,37]. Thus, the ID ensemble allows molecular recognition by providing protein surfaces capable of binding specific target molecules from the assembly of signaling complexes [35,36,37]. A variety of computational, biochemical, and biophysical methods have confirmed the IDP nature of the GR AF1 in recent years [24,25,26,27,38,39]. It has been proposed that the IDP nature of the GR AF1 allows it rapidly to ‘‘sample’’ its environment until appropriate binding partners are found [38,39]. Then, either by induced-fit or selective binding of a particular AF1 conformer, a high-affinity and more persistent interaction occurs between AF1 and the relevant coregulatory protein(s) [24,25,26,27,38,39]. The IDP regions/domains including GR’s NTD/AF1 promote molecular recognition primarily through unique combination of high specificity and low binding affinity with their functional binding partners, recognize and bind a number of biological targets, and create propensity to form large interaction surfaces suitable for interactions with their specific binding partners [40,41,42,43,44,45,46,47].

We have reported that several factors can influence AF1 secondary/tertiary structure formation, including binding protein partners, binding of the GR DBD to DNA, post-translational modifications such as site-specific phosphorylation in the NTD and in some circumstances, the type and concentrations of naturally occurring intracellular organic osmolytes [9,25,28,38,39]. We have also reported that such induced conformation in AF1 plays an important role in facilitating AF1’s interaction with specific coregulatory proteins and subsequent transcriptional activity [9,24,25,26,27,38,39]. The ID domains of many transcription factors have been shown to undergo a disorder-order transition upon interaction with binding partners that act as coregulators [28,35]. We have also shown that interaction of the AF1 with that partner at appropriate concentrations may cause AF1 to adopt higher secondary/tertiary structure that leads to stabilize AF1 structure [24,25,26,27,38,39]. For example, the TATA box binding protein (TBP) directly binds to the GR AF1 domain in vitro and in vivo and induces secondary/tertiary structure formation in AF1 such that TBP binding-induced folding in AF1 significantly enhances AF1’s interaction with other coactivators and subsequent AF1-mediated, GRE-driven promoter-reporter activity [38,39]. This phenomenon has now been reported for some other SHRs [26].

## 4. Role of Phosphorylation in the Regulation of Intrinsically Disordered AF1 Structure and Functions

Phosphorylation is an important post translational modification that regulates protein functions, including those of transcription factors in eukaryotic cells [48,49,50,51,52]. For transcription factors, phosphorylation can modulate their DNA binding affinity, interaction with components of the transcription initiation complex, and intracellular translocations [53,54,55]. Like many other transcription factors, the GR is a phospho-protein; consequently kinases can phosphorylate GR at multiple sites, leading to altered GR transcriptional activity [56,57,58,59,60]. Cell and tissue-specific GR functions are heavily regulated by specific kinases [61]. In the human GR, five serine residues have been identified [59]. All these known phosphorylation sites identified in human GR are found in the IDP NTD [10,59,60]. Three of them (S203, S211, and S226) are located within the AF1 [59].

Phosphorylation of the AF1’s core region has been shown to stabilize its structure, i.e. to shift the ensemble of conformers to a higher fraction containing structure [39]. Such phosphorylation is biologically relevant. We have shown that p38 in the MAPK pathways is a potent kinase for in vitro phosphorylation of S211 on the human GR [62,63]. Glucocorticoid treatment of CEM (human leukemic) cells induces the upstream kinase of p38, which phosphorylates and actives p38, which in turn, phosphorylates the GR, establishing a forward-acting functional loop. Because, in vitro and in vivo, p38 phosphorylates the GR at this specific site, we tested the relevance to GR function. The results showed that in transfected cells, the non-phosphorylatable S211A GR mutant was considerably less potent in inducing an AF1-mediated, GRE-driven reporter gene, and in driving GR-mediated apoptosis induced by a synthetic glucocorticoid, dexamethasone [64]. More general relevance to a range of lymphoid malignancies was found when we showed that in several unrelated malignant lymphoid cell lines, a greater proportion of p38 relative to other MAPKs corresponded to relative sensitivity to GR-driven apoptosis [65], Other reports suggest that phosphorylation may affect GR stability and thus alter transcriptional activity of the receptor [66]. We have also shown that site-specific phosphorylation of the ID AF1 leads to disorder-order conformational transition such that AF1’s interaction with other critical coregulatory proteins, and subsequent transcriptional activity are significantly enhanced [62,64]. Garabedian and co-workers have also demonstrated that site-specific phosphorylation in GR, particularly S211 and S226, play an important role in gene regulation by the GR, for which AF1 is a main player, as discussed above [36,59,67,68].

Several reports suggest that the state of GR phosphorylation affects its interactions with other proteins. TSG101, a component of the ESCRIT-I complex, has been reported to be preferentially recruited to the nonphosphorylated form of the GR [68]. It has been suggested that TSG101 stabilizes ligand-unbound GR in its unphosphorylated form to protect it from degradation. Thus, TSG101 interaction with GR may be important to keep unliganded GR protected from auto-degradation until the GR becomes hyper-phosphorylated. Interaction with DRIP150 (another GR coregulator and part of mediator complex) also has been reported to be modulated through GR phosphorylation [59]. Thus, it can be concluded that site-specific phosphorylation of the AF1 domain of GR can either enhance or diminish recruitment of coregulators, reflecting the biologic need for the GR to up- or down-regulate gene(s) in a cell- and promoter- specific manner by interactions with specific combinations of cofactors.

## 5. Discussion

Compared to protein segments with well-define globular structures, protein phosphorylation of Ser residue predominantly occurs within ID regions of signaling molecules [44,69,70,71]. This is significant because the formation of new hydrogen bonds would be more difficult if the sites of phosphorylation were located within ordered regions [36]. Thus, phosphorylation may regulate protein functions of the GR by affecting the conformational dynamics of the IDP NTD/AF1, leading to altered transcriptional activities [36,37,39,72]. As noted above, the GR exists in several translationally derived forms, successively shortened from the N terminus. The GR-C3 form is several times more active than the full-length, predominant “GRα” form [6,21,31]. It has been shown that this is due to loss of an NTD sub-domain, the R region, which exerts an allosterically repressive effect on GR’s AF1 function [31,73]. The structural and functional effects of site-specific phosphorylations of the several GR translational forms will be important to study.

The mechanisms by which GR controls gene expression pose a central problem in molecular biology, and the role of its ID AF1 is of immense importance. Phosphorylation elicits diverse effects on the biological functions of ID proteins by altering the energetics of their conformational landscape and by modulating interactions with other cellular components by stabilizing and/or inducing secondary structural elements [74,75,76,77]. There are also reports suggesting that in IDP receptors, poly-electrostatic interactions may also play important role [78]. Thus, signaling cascades that induce phosphorylation of the GR are important factors in determining the physiological actions of its ID NTD/AF1.

## 6. Summary and Perspectives

Glucocorticoids, working through the GR, regulate a variety of human physiological processes in a cell/tissue-dependent manner at the level of gene regulation. Glucocorticoids have also been frontline therapy for decades in the treatment of several pathological and disease conditions; however, the exact mechanism by which GR passes signals from ligand to regulate specific genes is not fully understood. Knowledge of 3-D structure of the full-length GR will of course be the starting point to provide answers to several questions on the actions of the GR. Post-translational modifications including phosphorylation, ubiquitination, and sumoylation have all been shown to affect functions of NHR family members. There is evidence that that differential phosphorylation stabilizes the structure of the GR’s IDP region and thus is a regulator of GR actions; yet it is also quite clear that the role of GR phosphorylation is a remarkably complex phenomenon.

Several outstanding questions remain to be answered: (1) What are the relative levels of phosphorylation of individual sites in specific cell/tissue- types under physiological conditions? (2) How does each phosphorylation site contribute to GR-mediated signaling through conformational rearrangements in the otherwise IDP AF1/NTD? (3) What are the allosteric consequences for the holo-GR? (4) Do the sequence of site-specific phosphorylations and the patterns of multiple-site phosphorylations matter? (5) What is the correlation between cell-based studies and in vivo animal models? (6) What are the effects of phosphorylations on the many GR isoforms?

**Figure 2:**
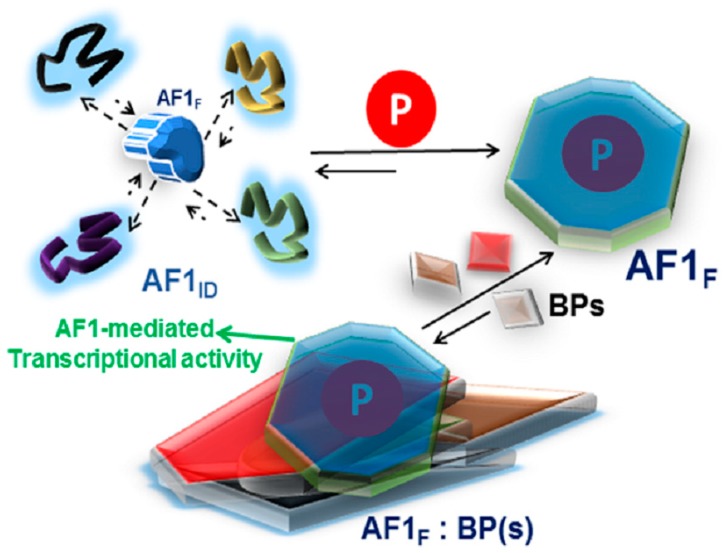
A proposed model of the effect of phosphorylation on the folding of intrinsically disordered (ID) AF1 domain of the glucocorticoid receptor. AF1 exists in equilibrium with mostly unstructured AF1_ID_ and a small fraction of folded (AF1_F_) conformers. Due to AF1’s site-specific phosphorylation (P), the equilibrium is shifted in favor of folded conformers. This structural rearrangement in AF1 creates surfaces well suited for interaction with coregulatory binding partner (BP) proteins (shown by different shapes and colors). The interaction with these BPs results in the regulation of AF1-mediated transcription of GR target gene(s). Based on references [62,72].

Based on studies from our laboratory and those of others, we propose that phosphorylation-induced conformational changes in the ID AF1 may be dependent upon the phosphorylation of individual site(s) such that the effects of one phosphorylated GR site may be influenced by the relative phosphorylation of other sites (Figure 2). Cell/tissue-specific effects of GR are tightly regulated through specific kinase(s)/phosphatase(s), and site-specific phosphorylation-induced conformational changes in ID NTD/AF1 and its subsequent effects on transactivation activities may provide critical information on how different surfaces within the ID AF1/NTD may be created and used to manipulate GR target gene expression. How site-specific phosphorylation leads to induced conformations in the ID AF1 and what kind of functional folded conformation it adopts in the full-length receptor are open questions.
